# Assessing the Influence of Cumulative *Chlorella vulgaris* Intake on Broiler Carcass Traits, Meat Quality and Oxidative Stability

**DOI:** 10.3390/foods13172753

**Published:** 2024-08-29

**Authors:** Ana R. Mendes, Maria P. Spínola, Madalena Lordelo, José A. M. Prates

**Affiliations:** 1 LEAF—Linking Landscape, Environment, Agriculture and Food Research Centre, Instituto Superior de Agronomia, Universidade de Lisboa, Tapada da Ajuda, 1349-017 Lisboa, Portugal; rmendes@isa.ulisboa.pt (A.R.M.); mlordelo@isa.ulisboa.pt (M.L.); 2 CIISA—Centro de Investigação Interdisciplinar em Sanidade Animal, Faculdade de Medicina Veterinária, Universidade de Lisboa, Av. da Universidade Técnica, 1300-477 Lisboa, Portugal; mariaspinola@fmv.ulisboa.pt; 3 Associate Laboratory for Animal and Veterinary Sciences (AL4AnimalS), Av. da Universidade Técnica, 1300-477 Lisboa, Portugal; 4 Associate Laboratory TERRA, Instituto Superior de Agronomia, Universidade de Lisboa, Tapada da Ajuda, 1349-017 Lisboa, Portugal

**Keywords:** microalgae, poultry, cumulative intake, carcass traits, meat quality, oxidative stability

## Abstract

The impacts of cumulative *Chlorella vulgaris* intake (proportion of microalga in the diet multiplied by the total feed consumed by each bird) on broiler carcass traits, meat quality and oxidative stability were reviewed to identify the optimal intake levels for maximising benefits. Our findings indicate that a cumulative intake of 8.73 g/bird significantly enhances thigh yield, while levels ranging from 8.73 to 401 g/bird optimise carcass weight and overall meat quality. However, higher cumulative levels may reduce carcass dressing percentage due to metabolic inefficiencies. Furthermore, *C. vulgaris* intake improves the oxidative stability of broiler meat by increasing antioxidant levels and balancing pro- and antioxidants. Including *C. vulgaris* in broiler diets boosts total carotenoid content, and antioxidant assays confirm that it enhances meat oxidative stability, with low to moderate cumulative intake levels (8.73 to 401 g/bird) providing the best balance of benefits. Optimal oxidative stability and antioxidant properties were observed at a cumulative intake level of 401 g/bird, showing significant improvements in meat antioxidant capacity. Higher levels may lead to diminishing returns or potential negative effects due to the digestibility issues of the microalga. Future research should refine intake models, understand the bioavailability of *C. vulgaris* nutrients and explore cost-effective methods to enhance its digestibility, to ensure its viability and sustainability as a feed additive.

## 1. Introduction

*Chlorella vulgaris* (*C. vulgaris*) was first described in 1890 by Martinus Beijerinck, a distinguished microbiologist and botanist [1]. With the world’s population projected to reach 9.7 billion by 2050, there is an urgent need for sustainable and health-enhancing livestock feeds [2]. Estimates indicate that maize and soybean, two of the main conventional feedstuffs for broiler production, will face unsustainable competition shortly due to their demand as human food, animal feed ingredients, and biofuel sources [3]. This challenge has led to an increased focus on natural additives and alternative feedstocks that can improve the well-being and productivity of livestock [4,5]. Microalgae have attracted considerable interest as feed supplements in poultry diets because of their potential to enhance the health and growth performance of broilers. Their nutritional benefits have made them a significant focus of research and study [6,7].

*Chlorella vulgaris*, in particular, is highly esteemed for its rich nutritional profile, including high concentrations of protein, vitamins (especially D and B12, which are typically absent in plant-based food sources), minerals and essential fatty acids [8,9]. This makes *C. vulgaris* an excellent candidate for improving the quality and sustainability of broiler feed. These nutrients contribute to enhanced growth rates, stronger immune responses and overall health in broilers. Research has shown that adding *C. vulgaris* to poultry diets can significantly improve the feed conversion ratio (FCR), carcass quality and various health indicators [6,10,11]. Thus, *C. vulgaris* emerges as a promising alternative to traditional feed additives, meeting the increasing demand for more sustainable and health-enhancing livestock feed options. *C. vulgaris* is also rich in bioactive compounds, such as chlorophylls, carotenoids and omega-3 (n-3) fatty acids [9,12,13,14,15], as well as essential amino acids [16]. These compounds not only serve as nutritional supplements but also significantly enhance the growth and health of broilers.

Additionally, *C. vulgaris* is renowned for its strong antioxidant properties [12]. These properties aid in mitigating oxidative stress, thereby protecting broiler cells from damage and supporting overall healthy physiological functions [10,11]. Antioxidant compounds, such as carotenoids and chlorophylls, are integral to this protective effect. Research has shown that supplementing broiler diets with *C. vulgaris* results in significant enhancements in growth performance and feed efficiency. Its high protein content and well-balanced amino acid profile promote muscle development and weight gain. For instance, the inclusion of *C. vulgaris* in the diet has been shown to improve final body weight and feed conversion ratio (FCR), indicating a more efficient conversion of feed into body mass [17]. Furthermore, supplementing with *C. vulgaris* enhances the nutritional quality of meat by increasing beneficial fatty acids and reducing harmful lipid oxidation, thus improving meat quality and consumer acceptability [5,18,19].

*Chlorella vulgaris* is abundant in proteins and various bioactive substances, including polysaccharides, polyphenols and pigments [20]. Compounds such as beta-glucans and other polysaccharides present in *C. vulgaris* play a role in modulating the immune system, thereby enhancing both innate and adaptive immunity in broilers. This results in increased antibody production, improved disease resistance and overall better health [9,11,12,21,22]. The antioxidant properties of the bioactive compounds in *C. vulgaris* help in reducing oxidative stress, thereby minimizing cellular damage and promoting healthy physiological functions [10,11,23]. Additionally, supplementing broiler diets with *C. vulgaris* has been linked to higher immunoglobulin levels, positioning it as a beneficial alternative to antibiotics that support productivity and animal welfare.

Incorporating *C. vulgaris* into broiler diets poses several challenges, particularly concerning optimal inclusion levels, feeding duration and the indigestibility of its cell walls in monogastric animals such as broilers. These factors significantly influence growth performance and health outcomes. While certain levels of *C. vulgaris* supplementation have shown promise in enhancing growth performance, variations in dosage and feeding duration can result in different health benefits and physiological responses. For instance, research suggests that low inclusion levels (up to 2% of the diet) can improve FCR without negatively impacting growth, whereas higher levels may not provide additional benefits and could potentially have adverse effects [10,19]. A major challenge is the indigestibility of the *C. vulgaris* cell wall in monogastric animals, due to its rigid structure made of sporopollenin-like biopolymers. This indigestibility can limit nutrient bioavailability within the microalga, reducing its efficacy as a feed supplement. To address this, techniques, such as mechanical disruption, enzymatic treatment, fermentation or pulse electric fields, are often employed to break down the cell walls and enhance nutrient availability, thereby increasing the complexity and cost of using *C. vulgaris* in broiler diets [6,24,25,26,27,28].

Meat quality and oxidative stability are closely linked to the balance between pro-oxidants and antioxidants in the meat. Oxidative stability refers to the meat’s ability to resist oxidative changes, which can lead to rancidity and spoilage [18,29]. This balance is crucial for maintaining meat quality, including colour, flavour and nutritional value. Antioxidants, such as carotenoids and vitamin E, play a significant role in enhancing oxidative stability by neutralizing free radicals and preventing lipid oxidation, mainly polyunsaturated fatty acids (PUFAs). In addition, it is well known that dietary supplementation with these antioxidants can improve meat quality by reducing oxidative stress and enhancing the antioxidative capacity of the meat [30,31].

The primary objective of this review was to search major reference databases (PubMed, NCBI; Web of Science, Clarivate Analytics; Scopus, Elsevier B.V.; and Google Scholar, Google LLC, Mountain View, CA, USA) to assess the dose–response relationship between various cumulative levels of *C. vulgaris* intake and its effects on key broiler carcass parameters, meat quality traits and meat oxidative stability. We hypothesized that the effects observed are due to the unique transfer kinetics of *C. vulgaris*’s bioactive compounds to the birds. Ultimately, this review aimed to identify the optimal dosage ranges of *C. vulgaris* that maximize broiler carcass traits, meat quality characteristics and oxidative balance. Additionally, we aimed to identify any potential thresholds beyond which *C. vulgaris* supplementation could provide lower outcomes or adverse effects on these key parameters.

## 2. Effects of Varying Cumulative Intake Levels of *Chlorella vulgaris* on the Broilers’ Carcass Traits

Table 1 provides a summary of the effects of varying cumulative intake levels of *C. vulgaris* on several carcass traits in broilers, including carcass dressing percentage, carcass weight, thigh yield, breast meat cooking loss and water holding capacity. The cumulative intake of *C. vulgaris* was calculated based on the proportion of microalga in the diet and the total feed consumed by each bird. The initial weights of the broilers in the studies varied from 40.03 to 109 g, covering very young broilers (1-day-old) and older ones (21-days-old). The percentage of *C. vulgaris* included in the feed ranged from 0.05% to 20%, with trial durations spanning from 14 to 41 days. Consequently, cumulative alga intake varied widely, from 1.40 to 718 g/bird. Lower inclusion levels (e.g., 0.05%, 0.10%) resulted in cumulative intakes of around 1.4 to 4.35 g/bird, while higher inclusion levels (e.g., 10%, 15%, 20%) led to significantly higher cumulative intakes, ranging from 176 to 718 g/bird. These variations highlight the diverse impacts of *C. vulgaris* supplementation on broiler carcass traits across different studies.

Table 2 presents the results of a regression analysis aimed at predicting dependent carcass variables based on cumulative *C. vulgaris* intake, as compiled in Table 1. To ensure statistical reliability, the analysis included only variables with three or more degrees of freedom (dof). The data analysis was performed using SPSS software (version 29.0, 2024) and involved various regression and curve estimation techniques. These techniques are compound, cubic, exponential, growth, inverse, linear, logarithmic, logistic, power, quadratic and sigmoid models. The primary focus was on the cumulative *C. vulgaris* intake as the independent variable influencing broiler carcass traits, meat quality and oxidative stability. Additional data on broilers’ carcass traits, for variables with less than three dof, are described in Table A1 of Appendix A.

The carcass dressing percentage, which measures the proportion of live weight resulting in the dressed carcass, showed variability with different levels of cumulative *C. vulgaris* intake. For instance, Cabrol et al. [10] reported the highest dressing percentage of 74.46% at a 10% inclusion level, corresponding to a cumulative intake of 401 g/bird. In contrast, lower inclusion levels such as 0.10% (4.35 g/bird) yielded a dressing percentage of 61.3% [32]. The inverse model demonstrated an inverse relationship between cumulative *C. vulgaris* intake and carcass dressing percentage (R^2^ = 0.711, *p* = 0.009), suggesting that as the cumulative intake of *C. vulgaris* increases, the carcass dressing percentage tends to decrease. The inverse model equation y = 73.444 − (48.944/x) indicates that higher levels of *C. vulgaris* supplementation might reduce the carcass dressing percentage due to possible metabolic or digestive inefficiencies in broilers at high intake levels. This finding underscores the importance of optimizing *C. vulgaris* intake levels to balance benefits without exceeding the birds’ physiological capacity to utilize the supplement effectively.

Carcass weight, a key measure of meat yield, showed a positive correlation with cumulative *C. vulgaris* intake. Broilers fed a diet with a 10% inclusion level (401 g/bird) had the highest carcass weight of 2099 g [10]. Conversely, a lower cumulative intake of 6.71 g/bird (0.2% inclusion) resulted in a carcass weight of 1416 g [33]. The cubic model best fits this relationship (R^2^ = 0.942, *p* = 0.006), demonstrating a significant positive correlation. The cubic model equation y = 1457.699 + 5.041x − 0.011x^2^ + 6.559×10^−6^x^3^ suggests that initial increases in *C. vulgaris* intake lead to substantial gains in carcass weight, but the rate of weight gain slows down at very high levels of intake, indicating diminishing returns. This model helps identify the optimal range of *C. vulgaris* supplementation for maximising carcass weight without encountering a plateau effect at higher dosages.

Thigh yield, an important measure of meat quality and quantity, ranged from 25.8% to 29.6% in the studies reviewed. Abou-Zeid et al. [32] observed the highest thigh yield of 29.6% with a cumulative intake of 8.73 g/bird (0.2% inclusion). The exponential model applied to these data would suggest a consistent increase in thigh yield with higher cumulative *C. vulgaris* intake, as indicated by the model’s significant correlation (R^2^ = 0.961, *p* = 0.003). However, the simplified equation, y = 29.195, implies that the exponential model predicts a constant thigh yield value of 29.195, regardless of the cumulative value of *C. vulgaris* intake. This reflects the supplement’s positive impact on muscle deposition, particularly in the thighs, making *C. vulgaris* a valuable additive for improving meat quality and quantity.

Breast meat cooking loss, an important indicator of meat quality, showed variation with different levels of *C. vulgaris* intake. Lower cooking loss percentages, which are desirable for meat quality, were observed with moderate inclusion levels, such as 12.6% at a cumulative intake of 3.52 g/bird [34]. However, the cubic model used to analyse this relationship (R^2^ = 0.252, *p* = 0.539) suggests a weak and statistically insignificant correlation. The cubic model equation y = 22.129 − 0.001x + 7.794×10^−5^x^2^ − 1.028 × 10^−7^x^3^ indicates that while there is some variation in cooking loss with changes in *C. vulgaris* intake, this trait is likely influenced by other factors beyond *C. vulgaris* supplementation. The low R^2^ value and high *p*-value suggest that factors such as genetics, overall diet composition and environmental conditions may also play significant roles in determining breast meat cooking loss.

Water holding capacity (WHC), a crucial quality trait that affects meat juiciness and texture, showed improvement with higher levels of *C. vulgaris* intake. The highest WHC recorded was 88.33% at a cumulative intake of 20.0 g/bird (0.60% inclusion) [33]. The relationship between cumulative *C. vulgaris* intake and WHC is best described by a sigmoid model, suggesting a positive trend. The model equation y = 4.435 − (0.329/x) describes an S-shaped curve, indicating that WHC improves gradually with increasing *C. vulgaris* intake up to a certain point before levelling off. The R^2^ value of 0.311 and *p*-value of 0.193 indicate a non-significant correlation, suggesting that while there is a positive trend, other factors may also influence WHC. This S-shaped trend highlights the importance of identifying an optimal range of *C. vulgaris* supplementation that maximizes WHC without incurring unnecessary costs or potential negative effects associated with excessive intake.

**Table 1:** Effects of varying cumulative intake levels of *Chlorella vulgaris* on the carcass traits of broilers.

Starting Weight and Age	Microalga (%) in Feed and Trial Duration (days) ^1^	Cumulative Microalga Intake (g/bird) ^2^	Carcass Traits					References
			Carcass Dressing (%)	Carcass Weight (g) ^3^	Thigh Yield (%)	Breast MEAT Cooking Loss (%)	Breast Meat Water Holding Capacity (%) ^4^	
45.1 g, 1 d-old ^5^	0.05%, 34 d	1.40	-	-	-	27.1	-	[35]
72.56 g, 4 d-old ^5,6^	0.10%, 31 d	3.52	-	-	-	12.56	73.49	[34]
45.1 g, 1 d-old ^5^	0.15%, 34 d	4.27	-	-	-	26.1	-	[35]
40.03 g, 1 d-old ^5^	0.10%, 41 d	4.35	61.3	1533	28.9	-	-	[32]
41.8 g, 1 d-old	0.20%, 41 d	6.71	70.78	1416	-	21.00	83.26	[33]
40.03 g, 1 d-old ^5^	0.20%, 41 d	8.73	63.2	1593	29.6	-	-	[32]
41.8 g, 1 d-old	0.40%, 41 d	13.0	69.79	1450	-	20.33	86.82	[33]
45.1 g, 1 d-old ^5^	0.50%, 34 d	14.1	-	-	-	26.5	-	[35]
41.8 g, 1 d-old	0.60%, 41 d	20.0	71.69	1553	-	21.66	88.33	[33]
788 g, 21 d-old ^5^	10%, 14 d	176	-		-	23.0	-	[18]
107 g, 5 d-old ^5^	10%, 34 d	401	74.46	2099	26.82	29.18	79.21	[10]
109 g, 5 d-old ^5^	15%, 34 d	561	73.11	1891	26.12	27.06	80.94	[10]
106 g, 5 d-old ^5^	20%, 34 d	718	72.58	1700	25.81	24.13	83.62	[10]

^1^ Slaughtering day was not considered for this calculation. ^2^ Percentage of microalgae in the diet multiplied by the total feed ingested per animal during the experiment. If cumulative feed intake (CFI) results were not available, the following estimation was made: CFI (g/bird) [10] = CFI (g/pen)/number of birds; CFI (g/bird) [18] = CFI (g/d/pen) × number of trial days/number of birds; CFI (g/bird) [34] = CFI (g/d/bird) × number of trial days. ^3^ An estimation was done when the carcass weight was not available: Carcass weight (g) ([10,32,33]) = dressing (%) × final body weight (g). ^4^ Water holding capacity (%) [33] = (500 − (water holding capacity (cm^2^) × 8.4)) × 0.2. ^5^ Male broilers. ^6^ Female broilers.

**Table 2:** Summary of predictive models for carcass traits based on cumulative *Chlorella vulgaris* intake.

Variable	Best Model Type	R-Square	Degrees of Freedom	*p*-Value	Model Equation
Carcass dressing (%)	Inverse	0.711	6	0.009	y = 73.444 − (48.944/x)
Carcass weight (g)	Cubic	0.942	4	0.006	y = 1457.699 + 5.041x − 0.011x^2^ + 6.559 × 10^−6^x^3^
Thigh yield (%)	Exponential	0.961	3	0.003	y = 29.195 × e^0.000x^ (simplified to y = 29.195)
Breast meat cooking loss (%)	Cubic	0.252	7	0.539	y = 22.129 − 0.001x + 7.794 × 10 − 5x^2^ − 1.028 × 10^−7^x^3^
Breast meat water holding capacity (%)	Sigmoid	0.311	5	0.193	y = 4.435 − (0.329/x)

Overall, these findings demonstrate that cumulative *C. vulgaris* intake significantly influences broiler carcass traits, with optimal intake levels crucial for maximising benefits. Inclusion levels of 0.2% (8.73 g/bird) enhance thigh yield, while 0.2% to 10% (ranging from 8.73 to 401 g/bird) optimise carcass weight and meat quality. For instance, a 0.10% inclusion (4.35 g/bird) yields a dressing percentage of 61.3%, whereas 10% (401 g/bird) results in a higher carcass weight of 2099 g. However, higher levels (e.g., 20%, 718 g/bird) may reduce carcass dressing percentage due to metabolic inefficiencies. Further research is necessary to refine intake models and understand the bioavailability of *C. vulgaris* nutrients, focusing on its bioactive compounds’ impact on broiler metabolism and health. This will help identify the optimal *C. vulgaris* intake levels for enhancing carcass traits and meat quality in broiler production, ensuring both efficiency and efficacy in its use as a feed additive.

## 3. Effects of Varying Cumulative Intake Levels of *Chlorella vulgaris* on the Broilers’ Meat Quality and Oxidative Stability

The microalga *C. vulgaris* has the potential to enhance meat quality by increasing levels of lipid-soluble antioxidant vitamins. These vitamins include vitamin E homologues (tocopherols and tocotrienols) and vitamin A, along with its precursors, such as certain carotenoids like β-carotene [36].

Table 3 provides a comprehensive overview of the effects of varying cumulative intake levels of *C. vulgaris* on meat quality traits in broilers, including breast meat pH 24 h post-slaughter (pH24h) and colour traits (L*, a* and b*). The cumulative microalga intake for these variables ranged from 1.40 to 718 g/bird. In addition, Table 4 presents the effects of varying cumulative intake levels of *C. vulgaris* on antioxidant (carotenoids) and pro-oxidant (PUFA) compounds, as well as oxidative stability indicators in broiler breast meat. The cumulative intake here ranged from 3.52 to 718 g/bird. The PUFA include linoleic acid (LA, 18:2n-6), alpha-linolenic acid (ALA, 18:3n-3), eicosapentaenoic acid (EPA, 20:5n-3), docosapentaenoic acid (DPA, 22:5n-3) and docosahexaenoic acid (DHA, 22:6n-3). Meat oxidative stability measures are the 2,2-diphenyl-1-picrylhydrazyl with free radical scavenger activity (DPPH free RSA) test, the ferric-reducing antioxidant power (FRAP) assay and the total phenolic content (TPC) method.

Table 5 presents a comprehensive summary of the regression analysis performed using SPSS software (version 29.0, 2024) to predict dependent variables associated with meat quality traits and oxidative stability, considering the cumulative intake of *C. vulgaris*. This analysis integrates data from Table 3 and Table 4, offering insights into how cumulative consumption of *C. vulgaris* influences these specific meat quality parameters and oxidative stability measures. To ensure statistical reliability, only variables with three or more dof were included in the analysis. Data were analysed as described before for carcass trait variables, using the cumulative intake of *C. vulgaris* as the independent variable influencing various meat variables metrics. The table details the best-fitting model type, R-square values, dof, *p*-values and the model equations for each variable. Data on broilers’ meat quality for variables with less than three dof are provided in Table A2 of Appendix A.

The breast meat pH24h, which indicates the acidity level of the meat and can influence meat quality and shelf life, varied with different levels of *C. vulgaris* intake. For instance, An et al. [35] reported a pH24h of 5.68 at a cumulative intake of 14.1 g/bird (0.50% inclusion), while higher inclusion levels such as 0.60% (20.0 g/bird) yielded a pH24h of 6.603 [30]. The regression analysis in Table 5 utilizes a sigmoid model (R^2^ = 0.163, *p* = 0.219), indicating a non-significant correlation between cumulative *C. vulgaris* intake and pH24h. The model equation y = 1.817 − (0.111)/x suggests a slight decrease in pH24h as *C. vulgaris* intake increases. However, the weak correlation implies that other factors significantly influence meat pH beyond *C. vulgaris* intake.

The lightness (L*) of breast meat, which measures the brightness of the meat, varied with different levels of *C. vulgaris* intake. For example, An et al. [32] reported the highest lightness value of 60.3 at a cumulative intake of 1.40 g/bird (0.05% inclusion). In contrast, a cumulative intake of 401 g/bird (10% inclusion) yielded a lightness value of 54.63 [6]. The cubic model (R^2^ = 0.909, *p* = 0.046) suggests a significant relationship between cumulative *C. vulgaris* intake and meat lightness. The model equation y = 60.123 − 0.163x + 0.001x^2^ − 4.626 × 10^−7^x^3^ indicates that lightness decreases with increasing *C. vulgaris* intake initially but can stabilize or even improve slightly at higher levels, reflecting the complexity of *C. vulgaris*’s impact on meat colour.

The redness (a*) of breast meat, which indicates the intensity of the red colour and is often associated with fresh meat quality, varied with different levels of *C. vulgaris* intake. Alfaia et al. [18] observed the highest redness value of 4.45 at a cumulative intake of 176 g/bird (10% inclusion). In contrast, lower inclusion levels, such as 0.15% (4.27 g/bird), resulted in a redness value of 0.57 [35]. The cubic model (R^2^ = 0.867, *p* = 0.079) suggests a tendential relationship between cumulative *C. vulgaris* intake and meat redness. The model equation y = 0.701 + 0.039x + 0.000x^2^ + 1.096 × 10^−7^x^3^ indicates that redness increases with higher *C. vulgaris* intake.

The yellowness (b*) of breast meat, which measures the intensity of the yellow colour and can significantly affect consumer perception, varied with different levels of *C. vulgaris* intake. Cabrol et al. [10] reported the highest yellowness value of 20.14 at a cumulative intake of 561 g/bird (15% inclusion). In contrast, a cumulative intake of 1.40 g/bird (0.05% inclusion) resulted in a yellowness value of 7.89 [35]. The cubic model (R^2^ = 0.998, *p <* 0.001) demonstrates a significant relationship between cumulative *C. vulgaris* intake and meat yellowness. The model equation y = 7.963 − 0.004x − 1.228 × 10^−7^x^3^ suggests that yellowness increases with higher *C. vulgaris* intake, indicating a substantial impact of the microalga on this colour trait.

Total carotenoid content in broiler breast meat, crucial for nutritional quality and antioxidant properties, showed a significant variation with different levels of *C. vulgaris* intake. The sigmoid model (R^2^ = 0.983, *p* = 0.008) suggests a significant relationship, with the model equation y = 7.922 − (458.036/x). This indicates a sharp increase in carotenoid content at lower *C. vulgaris* levels, plateauing at higher intakes. Despite the strong correlation, the limited number of dof (2) suggests caution is required in interpreting these results.

Regarding pro-oxidant fatty acids, LA, an essential n-6 PUFA, is critical for broiler meat quality. The sigmoid model (R^2^ = 0.730, *p* = 0.065) indicates a tendential significant correlation between *C. vulgaris* intake and LA content, with the model equation y = 3.323 − (1.701/x). LA content increases initially with *C. vulgaris* intake but levels off at higher levels. ALA, an essential n-3 PUFA, showed a non-significant correlation with cumulative *C. vulgaris* microalga intake. The sigmoid model (R^2^ = 0.310, *p* = 0.330) and the model equation y = 0.424 + (0.437/x) suggest a minimal increase in ALA content with lower *C. vulgaris* intake. The relationship is not statistically significant, indicating that random factors may largely influence ALA content. EPA, an n-3 PUFA known for its health benefits, showed a significant inverse relationship with *C. vulgaris* intake. The inverse model (R^2^ = 0.951, *p* = 0.005) and the model equation y = 0.205 + (2.693/x) indicate that EPA content decreases with increasing *C. vulgaris* intake. DPA, another n-3 PUFA, exhibited a significant exponential decrease with increasing *C. vulgaris* intake. The exponential model (R^2^ = 0.917, *p* = 0.043) and the model equation y = 0.500⋅e^−0.002x^ confirm this trend. However, the limited number of dof (2) suggests careful interpretation is necessary. DHA, an n-3 PUFA crucial for brain and heart health, showed a non-significant correlation with *C. vulgaris* intake. The logarithmic model (R^2^ = 0.176, *p* = 0.481) and the model equation y = −2.942 + 0.622ln*x* suggest a slight increase in DHA content with higher *C. vulgaris* intake, but the relationship is not statistically significant. Total PUFA content, essential for health benefits, showed a significant relationship with *C. vulgaris* intake. The sigmoid model (R^2^ = 0.963, *p* = 0.003) and the model equation y = 3.693 − (1.943/x) indicate a significant increase in PUFA content with increasing *C. vulgaris* intake, which levels off at higher intakes.

The antioxidant properties of *C. vulgaris* in broiler meat were evaluated using the DPPH assay, which measures the antioxidant capacity by evaluating free radical scavenging activity. El-Bahr et al. [34] reported a DPPH-free RSA of 17.11% at a cumulative intake of 3.52 g/bird (0.10% inclusion). At a much higher intake of 176 g/bird (10% inclusion), Alfaia et al. [18] observed a DPPH-free RSA of 25.8%. Further increasing the cumulative intake to 561 g/bird (15% inclusion) resulted in a DPPH-free RSA of 28.609%, with a slight decrease to 23.537% at 718 g/bird (20% inclusion) [6].

In addition to the DPPH assay, the FRAP assay, which measures the reducing power, showed that FRAP values increased with higher *C. vulgaris* intake. Cabrol et al. [10] reported FRAP values of 287.3 mg of gallic acid equivalents (GAE)/100 g of dry weight (DW) at 401 g/bird (10% inclusion), 414.09 mg GAE/100 g DW at 561 g/bird (15% inclusion) and 405.97 mg GAE/100 g DW at 718 g/bird (20% inclusion). Furthermore, TPC, known for its antioxidant properties, was also influenced by *C. vulgaris* intake. The same authors reported TPC values of 153.3 mg GAE/100 g DW at 401 g/bird (10% inclusion), 174.7 mg GAE/100 g DW at 561 g/bird (15% inclusion) and 174.3 mg GAE/100 g DW at 718 g/bird (20% inclusion). Overall, cumulative *C. vulgaris* intake enhances the oxidative stability of broiler meat by increasing antioxidant levels and balancing pro- and antioxidants. The inclusion of *C. vulgaris* in broiler diets boosts total carotenoid content, which protects meat from oxidative damage and improves shelf life and nutritional quality. Antioxidant assays (DPPH, FRAP and TPC) confirm that *C. vulgaris* enhances meat oxidative stability, although there is a threshold beyond which no additional benefits are observed. Data indicate that low to moderate inclusion levels of *C. vulgaris* (0.2% to 10%, or 8.73 to 401 g/bird) generally provide the best balance of benefits for oxidative stability and meat quality. Higher levels may lead to diminishing returns or potential negative effects. Moreover, optimal oxidative stability and antioxidant properties were observed at cumulative intake levels around 10% inclusion (401 g/bird), showing significant improvements in meat antioxidant capacity. This suggests that careful management of *C. vulgaris* intake can optimize broiler meat quality by leveraging its antioxidant potential.

## 4. Safety and Regulations

Several important considerations emerge when evaluating the safety precautions and regulatory aspects of using *C. vulgaris* when used as a feed additive or ingredient in broiler feeding. It is imperative that dietary *C. vulgaris* is safe, particularly when it is controlled for contaminants. Regulatory authorities like the European Food Safety Authority (EFSA) and the U.S. Food and Drug Administration (FDA) generally recognize *C. vulgaris* as safe, provided it is produced and processed under strict quality control measures.

Research suggests that contaminants from freshwater sources in *C. vulgaris* are typically present at amounts below detectable thresholds, reinforcing its safety profile. Ensuring that *C. vulgaris* is free from contaminants like toxic metals or pathogenic microorganisms is crucial, as these can pose significant health risks to both poultry and consumers. The possibility of bioaccumulation of these contaminants in broiler tissues, especially at higher levels of *C. vulgaris* intake, requires demanding and regular safety assessments. This underscores the requirement for well-established safety protocols in the industrial production and processing of *C. vulgaris* for animal feed. Ensuring proper cultivation and production conditions can keep contaminant levels, such as heavy metals and harmful microorganisms, within acceptable limits [37]. Maintaining the safety of *C. vulgaris* as a feed additive requires strict adherence to quality control measures, including regular toxicity analyses and monitoring of microcystin levels.

The regulatory framework for using *C. vulgaris* microalga in animal feed is complex and varies across different regions [38,39]. Adherence to local and international regulations concerning feed safety, allowable additive levels and labelling requirements is essential [40,41]. These regulations are designed to ensure the safety of animal feed additives and, consequently, the safety of animal-derived food products for human consumption. Regulatory standards are often updated based on new scientific discoveries and public health considerations. Ongoing research adds to a growing body of evidence that may inform regulators in revising and updating guidelines for the use of *C. vulgaris* in poultry diets.

Further investigation is needed to determine the long-term safety of *C. vulgaris*, especially when used at high incorporation levels and over extended feeding periods. Although short-term studies indicate beneficial effects, the long-term consequences for both animal and human health are not yet fully understood. This gap in knowledge underscores the necessity for ongoing research and monitoring to detect any potential adverse effects, including the cumulative effect of bioactive components in *C. vulgaris* on the health of animals and the safety of food.

Summing up, although *C. vulgaris* microalga provides notable health advantages as a poultry feed additive, its safe integration into broiler diets requires a holistic strategy. This strategy must include stringent quality control, compliance with emerging regulatory standards and continuous research into its long-term safety and effectiveness. These measures are crucial to guarantee that broiler meat enhanced with *C. vulgaris* is not only advantageous but also safe and in line with regulatory requirements, thereby sustaining consumer confidence and market sustainability.

## 5. Conclusions and Future Perspectives

In conclusion, these findings demonstrate that cumulative *C. vulgaris* intake significantly influences broiler carcass traits, meat quality and oxidative stability, with specific optimal intake levels maximising benefits. For carcass parameters and meat quality, cumulative intake levels of 8.73 g/bird enhance thigh yield, while levels ranging from 8.73 to 401 g/bird optimise carcass weight and overall meat quality. However, higher cumulative levels, such as 718 g/bird, may decrease the carcass dressing percentage due to metabolic inefficiencies.

In addition, cumulative *C. vulgaris* intake enhances the oxidative stability of broiler meat by increasing antioxidant levels and balancing pro- and antioxidants. The inclusion of *C. vulgaris* in broiler diets boosts total carotenoid content, protecting meat from oxidative damage and improving shelf life and nutritional quality. Antioxidant assays confirm that *C. vulgaris* enhances meat oxidative stability, with low to moderate cumulative intake levels from 8.73 to 401 g/bird providing the best balance of benefits. Optimal oxidative stability and antioxidant properties were observed around a cumulative intake level of 401 g/bird, showing significant improvements in meat antioxidant capacity. Higher levels may lead to diminishing returns or potential negative effects, likely due to indigestibility issues.

Future research should focus on refining intake models and understanding the bioavailability of *C. vulgaris* nutrients, particularly the impact of its bioactive compounds on broiler metabolism and health. This will help identify optimal intake levels that enhance carcass traits and meat quality. Additionally, studies should address the variability in optimal inclusion levels and potential adverse effects at higher doses, providing precise dosing guidelines. Long-term studies are necessary to assess the sustained effects of *C. vulgaris* on broiler health and productivity while elucidating the mechanisms through which its bioactive compounds influence growth performance, immune response and antioxidant capacity. Exploring cost-effective methods to enhance *C. vulgaris* digestibility, such as mechanical disruption, enzymatic treatment and fermentation, will further optimize its use in broiler production, ensuring its viability and sustainability as a feed additive.

## Figures and Tables

**Table 3:** Effects of varying cumulative intake levels of *Chlorella vulgaris* on meat quality traits of broilers.

Starting Weight and Age	Microalga (%) in Feed and Trial Duration (Days) ^1^	Cumulative Microalga Intake (g/Bird) ^2^	pH24h	Colour Traits ^3^			References
			Absolute Value	Absolute Value (CIELAB Scale)			
			(pH Scale)	L*	a*	b*	
45.1 g, 1 d-old ^4^	0.05%, 34 d	1.40	5.69	60.3	1.24	7.89	[35]
72.56 g, 4 d-old ^4,5^	0.10%, 31 d	3.52	5.86	-	-	-	[34]
45.1 g, 1 d-old ^4^	0.15%, 34 d	4.27	5.74	58.6	0.57	8.15	[35]
41.8 g, 1 d-old	0.20%, 41 d	6.71	6.480	-	-	-	[33]
41.8 g, 1 d-old	0.40%, 41 d	13.0	6.610	-	-	-	[33]
45.1 g, 1 d-old ^4,5^	0.50%, 34 d	14.1	5.68	58.9	0.87	7.86	[35]
41.8 g, 1 d-old	0.60%, 41 d	20.0	6.603	-	-	-	[33]
788 g, 21 d-old ^4^	10%, 14 d	176	5.77	44.1	4.45	9.96	[18]
107 g, 5 d-old ^4^	10%, 34 d	401	6.08	54.63	1.4	17.46	[10]
109 g, 5 d-old ^4^	15%, 34 d	561	6.06	54.87	0.83	20.14	[10]
106 g, 5 d-old ^4^	20%, 34 d	718	6.15	51.02	0.97	19.39	[10]

^1^ Slaughtering day was not considered for this calculation. ^2^ Percentage of microalgae in the diet multiplied by the total feed ingested per animal during the experiment. If cumulative feed intake (CFI) results were not available, the following estimation was made: CFI (g/bird) [10] = CFI (g/pen)/number of birds; CFI (g/bird) [18] = CFI (g/d/pen) × number of trial days/number of birds; CFI (g/bird) [34] = CFI (g/d/bird) × number of trial days. ^3^ Colour scale: a*—redness; b*—yellowness; L*—lightness. ^4^ Male broilers. ^5^ Female broilers.

**Table 4:** Effects of varying cumulative intake levels of *Chlorella vulgaris* on antioxidant and pro-oxidant compounds, and oxidative stability indicators in the broiler breast meat.

Starting Weight and Age	Microalga (%) in Feed and Trial Duration (Days) ^1^	Cumulative Microalga Intake (g/Bird) ^2^	Total Carotenoids (µg/100 g) ^3^	Fatty Acids (% Total Fatty Acid) ^4^						DPPH Free RSA ^5^ (%)	FRAP Test (mg GAE/ 100 g DW) ^6^	TPC (mg GAE/ 100 g DW) ^7^	References
				LA (18:2n-6)	ALA (18:3n-3)	EPA (20:5n-3)	DPA (22:5n-3)	DHA (22:6n-3)	Total PUFA				
72.56 g, 4 d-old ^8,9^	0.10%, 31 d	3.52	-	17.11	1.73	0.97	-	1.19	23.13	-	-	-	[34]
788 g, 21 d-old ^8^	10%, 14 d	176	202	25.8	1.58	0.27	0.481	0.270	38.20	-	-	-	[18]
107 g, 5 d-old ^8^	10%, 34 d	401	849.50	33.422	1.335	0.109	0.481	0.270	42.87	9.29	287.3	153.3	[10]
109 g, 5 d-old ^8^	15%, 34 d	561	1430.50	28.609	1.563	0.170	0.919	0.643	40.72	11.58	414.09	174.7	[10]
106 g, 5 d-old ^8^	20%, 34 d	718	1293.25	23.537	1.662	0.303	1.386	1.120	38.14	11.14	405.97	174.3	[10]

^1^ Slaughtering day was not considered for this calculation. ^2^ Percentage of microalgae in the diet multiplied by the total feed ingested per animal during the experiment. If cumulative feed intake (CFI) results were not available, the following estimation was made: CFI (g/bird) [10] = CFI (g/pen)/number of birds; CFI (g/bird) [18] = CFI (g/d/pen) × number of trial days/ number of birds; CFI (g/bird) [34] = CFI (g/d/bird) × number of trial days. ^3^ For Cabrol et al. [10], the estimation of total carotenoids (µg/100 g) was determined considering an average of 25% dry matter on breast meat. ^4^ Fatty acids: ALA—alpha-linolenic acid; DHA—docosahexaenoic acid; DPA—docosapentaenoic acid; EPA—eicosapentaenoic acid; LA—linoleic acid; PUFA—polyunsaturated fatty acids. ^5^ DPPH free RSA (mg GAE/100 g DW)—the 2,2-Diphenyl-1-picrylhydrazyl (DPPH) measures the antioxidant properties, using free radicals for assessing the potential of substances to serve as hydrogen providers or free radical scavengers (FRS), expressed in mg of gallic acid equivalents (GAE) per 100 g of dry matter. ^6^ FRAP test (mg GAE/100 g DW)—Ferric reducing antioxidant power (FRAP) assay, expressed in mg of gallic acid equivalents (GAE) per 100 g of dry matter. ^7^ TPC (mg GAE/100 g DW)—Total phenolic content (TPC), expressed in mg of gallic acid equivalents (GAE) per 100 g of dry matter. ^8^ Male broilers. ^9^ Female broilers.

**Table 5:** Summary of regression models predicting dependent meat quality traits and anti- and pro-oxidative compounds from cumulative *Chlorella vulgaris* intake.

Variable	Best Model Type	R-Square	Degrees of Freedom	*p*-Value	Model Equation
Breast meat pH24h	Sigmoid	0.163	9	0.219	y = 1.817 − (0.111/x)
Breast meat colour trait L*	Cubic	0.909	3	0.046	y = 60.123 − 0.163x + 0.001x^2^ − 4.626 × 10^−7^x^3^
Breast meat colour trait a*	Cubic	0.867	3	0.079	y = 0.701 + 0.039x + 0.000x^2^ + 1.096 × 10^−7^x^3^
Breast meat colour trait b*	Cubic	0.998	3	<0.001	y = 7.963 − 0.004x + 0.000x^2^ − 1.228 × 10^−7^x^3^ (simplified to y = 7.963 − 0.004x − 1.228 × 10^−7^x^3^)
Total carotenoids	Sigmoid	0.983	2 ^☨^	0.008	y = 7.922 − (458.036/x)
LA (18:2n-6)	Sigmoid	0.730	3	0.065	y = 3.323 − (1.701/x)
ALA (18:3n-3)	Sigmoid	0.310	3	0.330	y = 0.424 + (0.437/x)
EPA (20:5n-3)	Inverse	0.951	3	0.005	y = 0.205 + (2.693/x)
DPA (22:5n-3)	Exponential	0.917	2 ^☨^	0.043	y = 0.500⋅e^−0.002x^
DHA (22:6n-3)	Logarithmic	0.176	3	0.481	y = −2.942 + 0.622ln*x*
Total PUFA	Sigmoid	0.963	3	0.003	y = 3.693 − (1.943/x)

^☨^ Low number of degrees of freedom (<3). Colour scale: a*—redness; b*—yellowness; L*—lightness. Fatty acids: ALA—alpha-linolenic acid; DHA—docosahexaenoic acid; DPA—docosapentaenoic acid; EPA—eicosapentaenoic acid; LA—linoleic acid; PUFA—polyunsaturated fatty acids.

## Data Availability

No new data were created or analyzed in this study. Data sharing is not applicable to this article.

## Appendix A

**Table A1:** Effects of varying cumulative intake levels of *Chlorella vulgaris* on the broilers’ carcass traits.

Starting Weight and Age	Microalga (%) in Feed and Trial Duration (Days) ^1^	Cumulative Microalga Intake (g/Bird) ^2^	Abdominal Fat (%)	Breast Muscle Yield (%)	Left Breast Meat (%) ^3^	Leg Meat (%) ^3^	Wing Muscles (%)	Breast Meat				Leg Meat	References
								Thawing Loss (%)	Drip Loss (%)	Bound Water (%)	Plasticity (cm^2^)	Cooking Loss (%)	
45.1 g, 1 d-old ^4^	0.05%, 34 d	1.40	1.77	-	6.48	9.04	-	-	-	-	-	31.8	[35]
72.56 g, 4 d-old ^4,5^	0.10%, 31 d	3.52	-	-	-	-	-	5.20	-	-	-	-	[34]
45.1 g, 1 d-old ^4^	0.15%, 34 d	4.27	2.01	-	6.48	9.30	-	-	-	-	-	32.7	[35]
40.03 g, 1 d-old ^4^	0.10%, 41 d	4.35	2.7	-	-	-	-	-	-	-	-	-	[32]
41.8 g, 1 d-old	0.20%, 41 d	6.7	-	-	-	-	-	-	-	58.25	2.690	-	[33]
40.03 g, 1 d-old ^4^	0.20%, 41 d	8.73	2.68	-	-	-	-	-	-	-	-	-	[32]
41.8 g, 1 d-old	0.40%, 41 d	13.0	-	-	-	-	-	-	-	61.81	2.656	-	[33]
45.1 g, 1 d-old ^4^	0.50%, 34 d	14.1	1.95	-	6.39	9.14	-	-	-	-	-	34.4	[35]
41.8 g, 1 d-old	0.60%, 41 d	20.0	-	-	-	-	-	-	-	63.32	3.210	-	[33]
788 g, 21 d-old ^4^	10%, 14 d	176	-	-	-	-	-	-	-	-	-	27.2	[18]
107 g, 5 d-old ^4^	10%, 34 d	401	-	25.11	-	-	7.24	3.43	2.17	-	-	-	[10]
109 g, 5 d-old ^4^	15%, 34 d	561	-	24.67	-	-	7.38	3.96	1.98	-	-	-	[10]
106 g, 5 d-old ^4^	20%, 34 d	718	-	24.39	-	-	7.38	4.04	1.92	-	-	-	[10]

^1^ Slaughtering day was not considered for this calculation. ^2^ Percentage of microalgae in the diet multiplied by the total feed ingested per animal during the experiment. If cumulative feed intake (CFI) results were not available, the following estimation was made: CFI (g/bird) [10] = CFI (g/pen)/number of birds; CFI (g/bird) [18] = CFI (g/d/pen) × number of trial days/number of birds; CFI (g/bird) [34] = CFI (g/d/bird) × number of trial days. ^3^ Left breast and thigh meats without skin and bones. ^4^ Male broilers. ^5^ Female broilers.

**Table A2:** Effects of varying cumulative intake levels of *Chlorella vulgaris* on meat quality variables in broiler meat.

Starting Weight and Age	Microalga (%) in Feed and Trial Duration (Days) ^1^	Cumulative Microalga Intake (g/Bird) ^2^	Breast Meat												Leg Meat				References
			Texture Profile Analysis ^3^				Sensory Traits ^4^					Chemical Composition			Colour Traits ^5^			pH24h	
												Protein (%)	Fat (%)	Cholesterol (mg/g)	Absolute Value (CIELAB Scale)			Absolute Value	
			Ce	H	S	Co	T	J	F	OF	OA				L*	a*	b*	(pH Scale)	
45.1 g, 1 d-old ^6^	0.05%, 34 d	1.40	-	-	-	-	-	-	-	-	-	-	-	-	55.5	2.84	9.01	5.87	[35]
45.1 g, 1 d-old ^6^	0.15%, 34 d	4.27	-	-	-	-	-	-	-	-	-	-	-	-	52.7	3.47	7.12	5.94	[35]
45.1 g, 1 d-old ^6^	0.50%, 34 d	14.1	-	-	-	-	-	-	-	-	-	-	-	-	54.4	3.06	8.63	5.86	[35]
788 g, 21 d-old ^6^	10%, 14 d	176	-	-	-	-	5.76	4.45	4.44	0.203	5.27	-	0.97	0.59	48.7	8.23	12.0	5.87	[18]
107 g, 5 d-old ^6^	10%, 34 d	401	14.16	28.09	0.77	0.62	-	-	-	-	-	-	-	-	-	-	-	-	[10]
107 g, 5 d-old ^6^	10%, 34 d	401	-	-	-	-	-	-	-	-	-	25.56	1.95	0.4067	-	-	-	-	[11]
109 g, 5 d-old ^6^	15%, 34 d	561	12.31	25.99	0.76	0.64	-	-	-	-	-	-	-	-	-	-	-	-	[10]
109 g, 5 d-old ^6^	15%, 34 d	561	12.31	25.99	0.76	0.64	-	-	-	-	-	27.1	0.92	0.4300	-	-	-	-	[11]
106 g, 5 d-old ^6^	20%, 34 d	718	13.17	26.91	0.77	0.61	-	-	-	-	-	-	-	-	-	-	-	-	[10]
106 g, 5 d-old ^6^	20%, 34 d	718	13.17	26.91	0.77	0.61	-	-	-	-	-	26.89	1.22	0.3900	-	-	-	-	[11]

^1^ Slaughtering day was not considered for this calculation. ^2^ Percentage of microalgae in the diet multiplied by the total feed ingested per animal during the experiment. If cumulative feed intake (CFI) results were not available, the following estimation was made: CFI (g/bird) [10] = CFI (g/pen)/number of birds; CFI (g/bird) [18] = CFI (g/d/pen) × number of trial days/number of birds; CFI (g/bird) [34] = CFI (g/d/bird) × number of trial days. ^3^ Sensory traits: Ce—chewiness; Co—cohesiveness; H—hardness (N); S—springiness. ^4^ Sensory traits: F—flavour; J—Juiciness; OA—overall acceptability; OF—off-flavour; T—tenderness. ^5^ Colour traits: L*—lightness; a*—redness; b*—yellowness. ^6^ Male broilers.
